# Deep Learning for Cell Migration in Nonwoven Materials and Evaluating Gene Transfer Effects following AAV6-ND4 Transduction

**DOI:** 10.3390/polym16091187

**Published:** 2024-04-24

**Authors:** Ilya I. Larin, Rimma O. Shatalova, Victor S. Laktyushkin, Stanislav A. Rybtsov, Evgeniy V. Lapshin, Daniil V. Shevyrev, Alexander V. Karabelsky, Alexander P. Moskalets, Dmitry V. Klinov, Dimitry A. Ivanov

**Affiliations:** 1 Center for Translational Medicine, Sirius University of Science and Technology, Krasnodar Territory Sirius, 1 Olympic Ave., Sirius 354340, Russia; 2 Resource Center for Cell Technology and Immunology, Sirius University of Science and Technology, Krasnodar Territory Sirius, 1 Olympic Ave., Sirius 354340, Russia; 3 Federal Research and Clinical Center of Physical-Chemical Medicine, Federal Medical Biological Agency, Moscow 119435, Russia; 4 Center for Genetics and Life Sciences, Sirius University of Science and Technology, Krasnodar Territory Sirius, 1 Olympic Ave., Sirius 354340, Russia; 5 Institut de Sciences des Matériaux de Mulhouse—IS2M, CNRS UMR 7361, F-68057 Mulhouse, France

**Keywords:** electrospun, mats, neural network, fibroblasts, transduction, AAV, cell homing and migration

## Abstract

Studying cell settlement in the three-dimensional structure of synthetic biomaterials over time is of great interest in research and clinical translation for the development of artificial tissues and organs. Tracking cells as physical objects improves our understanding of the processes of migration, homing, and cell division during colonisation of the artificial environment. In this study, the 3D environment had a direct effect on the behaviour of biological objects. Recently, deep learning-based algorithms have shown significant benefits for cell segmentation tasks and, furthermore, for biomaterial design optimisation. We analysed the primary LHON fibroblasts in an artificial 3D environment after adeno-associated virus transduction. Application of these tools to model cell homing in biomaterials and to monitor cell morphology, migration and proliferation indirectly demonstrated restoration of the normal cell phenotype after gene manipulation by AAV transduction. Following the 3Rs principles of reducing the use of living organisms in research, modeling the formation of tissues and organs by reconstructing the behaviour of different cell types on artificial materials facilitates drug testing, the study of inherited and inflammatory diseases, and wound healing. These studies on the composition and algorithms for creating biomaterials to model the formation of cell layers were inspired by the principles of biomimicry.

## 1. Introduction

The field of biomedical material research plays a crucial role in the progress of modern high-tech medicine [1]. The demand for these materials is rising due to an ageing population, increased risks of developing senile diseases, and the spread of such civilisational diseases as diabetes mellitus, osteoporosis, and cardiovascular diseases. All of this escalates the prevalence of chronic trophic ulcers, impairs wound healing, and increases the risk of surgical interventions and injuries [2,3].

**Figure 1:**
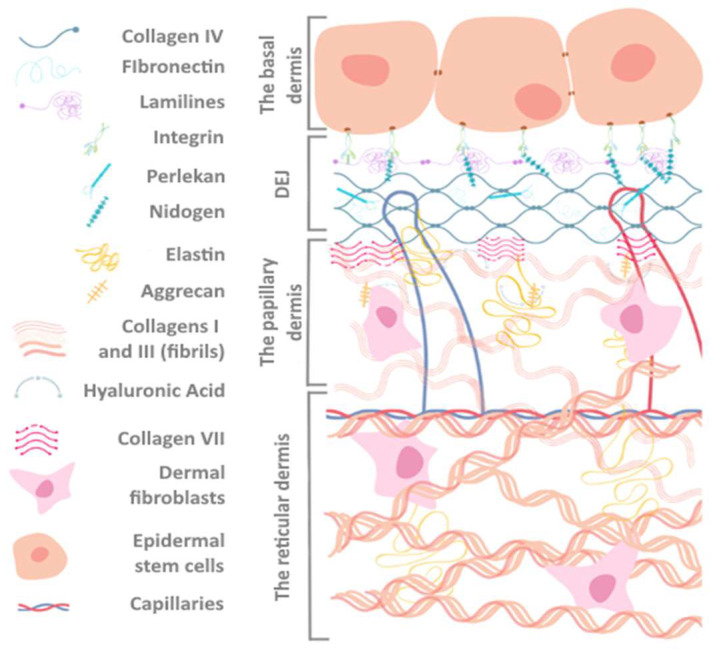
The multilayered arrangement of the main components of the dermoepidermal junction (DEJ) and dermis demonstrates the interaction of cell populations and the extracellular matrix (ECM).

In preclinical research, the most promising strategy for wound healing is the development of methods to generate an extracellular matrix (ECM) [4]. Fibroblasts, as the largest cell population in the dermis, are critical for maintaining the composition and stability of the ECM. They regulate the production and degradation of collagen, elastin, and other ECM components. On the other hand, the extracellular matrix has the ability to control the growth and movement of dermal fibroblasts and also affect their transdifferentiation into various cell types, influencing both the function and morphology of fibroblasts (see Figure 1) [5].

Functional polymer materials with unique properties have the potential to improve medical devices and drugs, leading to more effective treatments [6]. These novel polymers are being produced in biomedical research using a simple and technologically advanced electrospun process [7]. The morphology and proliferation of eukaryotic cells depend on their microenvironment, as the local orientation of the electro spun fibres has a pronounced influence on cells [8].

Artificial scaffolds provide a reliable surface for cell attachment, ECM formation, transdifferentiation, and functional tissue formation. Scaffolds can be created from different materials, which include natural, synthetic, or composite materials. Techniques such as pore-forming leaching, electrospinning, molecular self-assembly, and phase separation can be used to create these scaffolds. A potential solution for overcoming specific limitations of individual materials, such as inadequate mechanical properties, weak cell adhesion, and inappropriate biodegradation rates, is the use of composite biomaterials [9]. Electrospun materials offer significant benefits for tissue engineering due to their ability to closely imitate natural scaffold structures as well as sustain cell migration and proliferation. Dermis scaffold implants accelerate the healing process of partially or completely damaged skin caused by thermal or chemical exposure [10]. Furthermore, the three-dimensional scaffold matrix promotes tissue regeneration while preventing the formation of new wound lesions during the initial healing stages. The low cytotoxicity and adhesive properties of the scaffold’s structural features facilitate cell migration along the fibres within the wound. Thus, the key feature of nonwoven polymer materials in medical applications is their ability to support cell migration and homing for tissue formation. For materials scientists in biotechnology, the establishment of reliable methods for assessing cell migration, proliferation, and homing is a challenging task.

Operando research in biomaterials requires advanced imaging techniques, real-time monitoring tools, and specialised experimental set-ups to observe and analyse materials in their functional states in natural environments [11].

The migration of fibroblast cells is pivotal in the wound recovery process, bone formation, and implant healing. The application potential and utility of nanofibre mats in tissue engineering will be maximised if they promote cell monolayer and ECM formation. Model materials such as PLA-gelatine are useful for extending and modifying the fibre surface topology. Convolutional or deep neural network (CNN) analysis, by monitoring nuclei as an indicator of cell migration, differentiation, and homing in different microenvironments, influences gelatine release as an important factor for cellular colonisation of mats. By cell migration, we mean the change in the number of cells in the volume of an artificial, synthetic, or native implant per unit of time and per unit of area. The proposed CNN model effectively assesses cellular stress based on migration rates, homing, and nuclear morphology. We have used primary fibroblasts with a mutation in the ND4 gene, which is used as a cell model for Leber Hereditary Optic Neuropathy (LHON) [12]. These fibroblast cells were previously used to generate iPSCs that differentiated along retinal ganglion cells (RGCs) [13]. In this way, we are creating disease models and drug development for gene therapy that exploit allotopic expression of the ND4 gene through MTS signalling [14,15]. We aimed to investigate whether the migration and homing of ND4 mutated primary fibroblasts would be altered after transduction by a therapeutic AAV vector compared with a GFP-expressing control. This is important because AAV vector transduction has been reported to be toxic to cells [16] and may induce cellular stress by activating the antiviral immune response.

## 2. Materials and Methods

### 2.1. Electrospun Technique

Poly-(d,l)-lactide (PLA, Ingeo 4032d, NatureWorks, 650 Industrial Park Drive, PO Box 564 Blair, NE 68008, USA) -gelatine (ServiceBio, Wuhan, China) solutions of 100 mg/mL and 200 mg/mL of 1,1,1,3,3,3-hexafluoro-2-propanol (HFiP, 105228-2KG, Sigma-Aldrich, St. Louis, MO, USA), respectively. The PLA-gelatine solutions were composed through combination at 1:1 and 1:3 vol. % ratios. For instance, mat A is PLA-gelatin (100 mg/mL) (3:1), mat B is PLA-gelatine (100 mg/mL) (1:1), mat C is PLA-gelatine (200 mg/mL) (3:1), and mat D is PLA-gelatine (200 mg/mL) (1:1). Electrospinning was conducted with an acceleration voltage of 30 kV and a flow rate of 1 mL/h using an HSW NORM-JECT syringe (Henke Sass Wolf, Noerten-Hardenberg, Germany) at a distance of 30 cm from the counter electrode on a MECC NF 500 (MECC CO, Kyoto, Japan) at a temperature of 25 °C and 45–55% humidity. A 22 gauge stainless steel needle was employed as the nozzle, and an emitting electrode of positive polarity, alongside a ground electrode, was connected to a flat static collector. A polymethylmethacrylate (PMMA) dielectric collector was utilised to acquire freestanding mats positioned 10 cm from the counter-electrode. The mats that were 150–300 μm thick were deposited into Petri dishes 9 cm in diameter (Perint, St. Petersburg, Russia). Subsequently, the dishes were incubated for 24 h at 30 °C.

### 2.2. Scanning Electron Microscopy

The surface morphology and topography of the electrospun nanofibres, as well as morphology of the primary fibroblasts, underwent examination via a scanning electron microscope (Carl Zeiss Crossbeam 550, Oberkochen, Germany). The SEM was operated at an accelerating voltage of 8 kV and a current of 150 pA. A Quorum (Q150T S/E/ES+, London, UK) was used to deposit a 10 nm gold-palladium (80:20 at.%) coating. Around 100 random measurements using ImageJ image analysis software (National Institutes of Health, Bethesda, MD, USA) were taken to estimate the diameter and distribution [17]. Two detectors such as SE2 and InLens were employed to measure the cell area. The SE2 detector is apt for visualising the cell surface topology and cell boundaries, while the InLens detector allows the outline of a cell’s nucleus to be visible. The cell morphology was analysed by quantifying the cell area and cell proliferation.

The primary human skin fibroblasts were prepared for SEM through three steps: fixation, dehydration, and plating. The cells were fixed to a 4% formalin solution for 30 min at room temperature and then washed twice with DPBS. The fixed cells on the mat holder were subjected to stepwise exposition in 500 μL ethanol-water solution for 5 min at 10%, 20%, 30%, 40%, 50%, 60%, 70%, 70%, 80%, 90%, and 96% (the 70% and 96% steps were prolonged to 10 min) [18]. The samples were incubated in a 1:1 mixture of hexamethyldisilazane (HMDS)-ethanol for 10 min and subsequently subjected to three rounds of incubation in 100% HMDS (300 μL). The first two incubations lasted for 10 min each, while the third incubation was extended overnight to ensure complete evaporation of the HMDS.

### 2.3. Dynamic Mechanical Analysis

Dynamic mechanical analysis (DMA) was carried out using an RSA-G2 (TA Instruments, New Castle, DE, USA). The test was performed at a constant tensile rate of 5 × 10^−3^ mm/min in accordance with ASTM D638 without any preload until the sample broke [19]. The measurements were taken at a temperature of 37 °C. The samples had an aspect ratio of 1:6 and a thickness that varied from 150 to 300 μm. Determining the mechanical properties of thin nanofibre composites is challenging due to the limitations of anchorage and the application of excessive strain. These samples were incubated at 37 °C in dPBS for a fixed period of time. They were mounted in a special frame to avoid mechanical deformation. We tested at least three samples from each batch of electrospun mats.

### 2.4. Laser Scanning Confocal Microscopy

Primary fibroblast proliferation and migration were analysed after 1, 4, and 7 days. An LSM 980 confocal laser scanning microscope (Carl Zeiss, Oberkochen, Germany) with a Plan-Apochromat 20×/0.8 objective was used to assess proliferation and cell migration. The laser power at 405 nm was set to 0.5–1% nominal power. The cells were fixed with a 4% solution of buffered cold formalin for 15 min, followed by washing twice with DPBS. Then, the cell nuclei were stained with Hoechst 33342 (Thermo Fisher Scientific, Waltham, MA, USA) and propidium iodide.

### 2.5. AAV Production

Briefly, a suspension of HEK293 cells (ECACC 85120602) was transfected with pAAV expression plasmid (ND4 or GFP), pHelper Vector (Part No. 340202, Cell Biolabs Inc., San Diego, CA, USA), and pAAV-RC6 Vector (Part No. VPK-426) with a molar ratio of 2:2:5, respectively. Then, pAAV-GFP plasmid (Cat. number VP-401, CellBiolabs, San Diego, CA, USA) was used to produce AAV6-GFP vector. To produce an AAV vector for allotopic expression of ND4, the pAAV-COX8-ND4 plasmid, expressing the ND4 gene with a COX8 mitochondrial localisation signal at the 5′ end under the control of the CMV promoter, was used (Supplementary Figure S1). DNA (1.5 µg per 1 million cells) was mixed with PEI MAX (Linear polymer, MW 40’000, Polysciences Inc., Warrington, PA, USA) at a mass ratio of 1:5, respectively, to a final volume of 5%. After transfection, the cells were cultivated in BalanCD HEK293 medium (Irvine Scientific, Santa Ana, CA, USA) at 37 °C, 5% CO_2_, and 100 rpm for 120 h. The cells were then lysed (0.05% Tween-20), nuclease-treated, and centrifuged at 3000× *g* for 10 min, and the supernatant was filtered by 0.22 μm membranes. The concentrate was then purified by affinity chromatography (AAVX, Thermo Fisher Scientific). The number of viral genomes was determined by qPCR. The obtained sample was subsequently filtered by a 0.22 μm syringe filter (TPP 99722).

### 2.6. Fibroblast Transduction by AAV

The fibroblasts were transduced with 30,000 viral genomes per cell (MOI), and 500,000 cells were transferred to 3 cm TC-treated plates. After 17–18 h of incubation (37 °C, 5% CO_2_), the medium was collected, the cells were trypsinised according to the protocol described above, and after 36 h, the transduced cells were plated onto the mats. The efficiency of transduction was estimated by GFP expression and reached 50% GFP+ cells after 17–18 h of culturing by fluorescence intensity on Axio Vert.A1 (Carl Zeiss, Germany).

### 2.7. Convolutional Neural Network (CNN)

The U-Net [20] trained on the primary fibroblasts on the mats (5140 images) and the DSB dataset [21] detected nuclei in the PNG files using predefined metrics like the IoU and dice loss. Augmentation boosts the dataset in such a manner that reproducing raw images similar to the original produces a more robust model with a lower overfitting risk or better generalisation capabilities [22]. The intersection over union (IOU) can help assess a model’s performance on training and validation datasets for nuclei vision tasks. It shows whether the model can effectively generalise and be applied to new data. Fibroblast nuclei masks were manually designed for the model training. A training IOU is a metric on a training dataset that indicates the model’s segmentation quality of objects in the images used in training. A high training IOU demonstrates that the model is proficiently trained and efficiently identifies the objects in the images. A validation IOU is a metric value assigned to a validation dataset that is not utilised for model training. 

All testing and training of the neural network were carried out using an NVIDIA RTX 3060 graphics card (256 bits, 8 GB memory). Our study utilised two separate cell datasets comprising microscopic images from distinct biological fields. The first dataset consisted of 108 CZI files, each containing 14 layers and boasting a resolution of 13,926 × 13,926 ppi (Figure 4a). 

## 3. Results and Discussion

### 3.1. Mechanical Properties of Microenvironment

**Figure 2:**
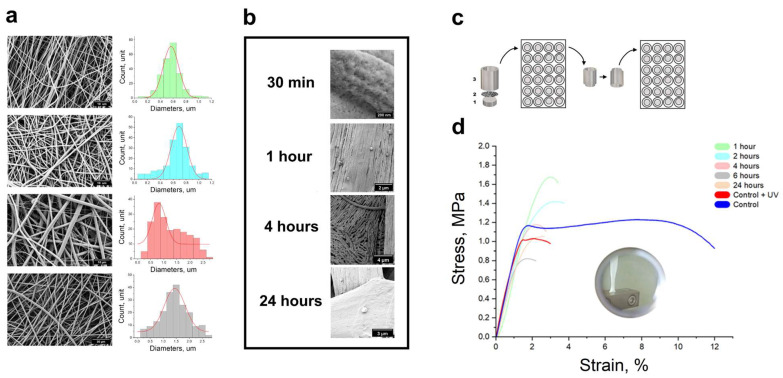
Compositions A, B, C, and D (from top to bottom). (**a**) SEM images of microstructure and diameter distribution of fibres. (**b**) Release of gelatine from fibres. (**c**) Fibrous mat-mounted cell culture with 1 cm^2^ inner diameter for PMMA holders. (**d**) Stress–strain curves for mat composition B.

The SEM images (Figure 2a) showed a dense layer of composite fibres on the mats. The average fibre diameter of the mats was 566 ± 123 nm for mat A, 690 ± 124 nm for mat B, 820 ± 255 nm for mat C, and 1440 ± 400 nm for mat D. The average diameter and standard deviation of a minimum of 200 fibres per sample are presented. Higher concentrations of gelatine in the fibres showed an irregular morphology, with more ribbons and thinner spider-like or reticular structures. Fibre diameter reduction was a variable parameter and was not investigated further.

The fibres underwent considerable topographical changes after being incubated in an aqueous medium for 30 min. The initial roughness of the fibre was substituted with deep furrows. Moreover, the surfaces of thin tapes were separated into plates, which combined with the fibres.

As the incubation time increased, grooves resembling cracks emerged on the fibres, which were still visible even after 24 h (Figure 2b). Images (Figure 2b) are provided for visual assessment of the fibre topography at 200 nm, 2 µm, 4 µm, and 3 µm magnifications after the 24 h incubation period. These observations are an indication that PLA-gelatine mats are readily subject to prolonged degradation. This allows for a positive prediction of its potential for long-term incubation in cell culture. The degradation level of the mats should match the rate of new tissue formation, ensuring optimal conditions for successful regeneration. Rapid mat degradation can inhibit cell proliferation, while slow mat degradation can hinder the restoration of biological tissue function [23]. Therefore, maintaining a balance between the rate of mat degradation and new tissue formation is critical.

The material must have adequate strength to resist manipulation during implantation and preservation under both storage and physiological conditions. The skin’s tensile strength is approximately 1–27 MPa [22] during the first and second stages of deformation, which promotes wound healing at the microscopic level, with mechanical signalling being critical for cell growth, transdifferentiation, and ultimately tissue engineering. The mechanical properties of the mats decreased from 1.6 MPa to 0.7 MPa as the strain decreased from 3% to 1.5% due to gelatine release during incubation (as presented in Figure 2d). This was accompanied by a reduction in fibre diameter [Table 1]. The tensile strength of the samples that were sterilised with UV radiation before cell seeding decreased to 0.8 MPa. UV irradiation can break down polymer chains into smaller fragments. During the experiment, the samples were subjected to a 14 W/cm^2^ ultraviolet lamp. Following 24 h of incubation, the mechanical properties of the samples remained soft and within the range of the native tissue strength.

### 3.2. Proliferation and Changes in Nuclear Morphology during Cell Migration and Homing

**Figure 3:**
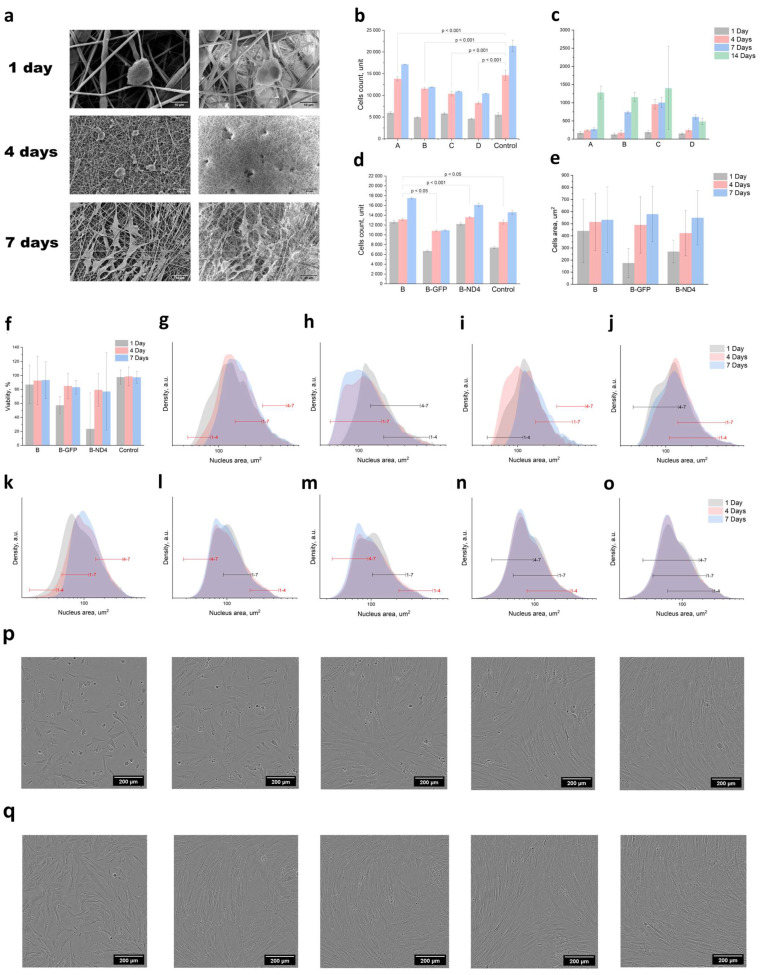
(**a**) SEM images of the morphology of the primary fibroblasts on mat B, cultured for 7 days. (**b**) Cell numbers on different mats through DL (2-SE; *p* values established by Freedman comparison). (**c**) Cell area per mat under SEM (SD). (**d**) Number of AAV-transduced GFP and ND4 cells through DL (2-SE; *p* values established by Freedman comparison). (**e**) Cell area per transduced cell on mat B under SEM (SD). (**f**) Cell viability (Kolmogorov test). (**g**–**j**) Cell nucleus area on the culture plate, mat B, transduced GFP, and ND4 (from left to right, with mean difference from Tukey comparison (red = significant)). (**k**–**o**) Cell nucleus area on the culture plate and mats A, B, C, and D (from left to right, with mean difference from Tukey comparison (red = significant)). (**p**) Cells without transduction (1–5 days). (**q**) Transduced cells (1–5 days). All parameters showed statistical significance (*p* < 0.05).

The viability of primary cells declines after a few passages in vitro, which is a disadvantage compared with immortalised cells [24]. However, primary human skin fibroblasts can retain characteristics identical to cells of intact tissue over several passages. Primary fibroblasts are better suited to closely mimicking real-life processes and conditions, including cell migration and regeneration. We used a fibroblast culture that resembled intact tissue human fibroblasts regarding their morphological features and proliferation rate (Figure 3a). The fibroblasts without transduction (Figure 3p) and with GFP transduction (Figure 3q) are shown on a culture plate (imaging by Incucyte S3, Sartorius, Germany).

Nonwoven materials facilitate cell attachment and cell division and guide cell migration and differentiation, providing the necessary conditions for tissue engineering [25]. As previously found, the AAV6 serotype effectively transduces primary cell cultures, including fibroblasts and keratinocytes [26]. The experiments were carried out in several stages: assessment of cell proliferation and migration on nonwoven materials, selection of the best nonwoven material according to these parameters, and transduction of cells with AAV-GPP or AAV-ND4 to assess migration and proliferation on the selected nonwoven substrate. Analysis of the cell morphology is of particular interest, since changes in cell shape can be a sign of a change in the fibroblast phenotype (Figure 3a). We observed that on the fourth day of the experiment, on all mats, the cells acquired a spindle-shaped appearance and multidirectional filopodia.

On the electrospun mats, the proliferative activity of the primary fibroblasts was assessed on days 1, 4, and 7 of the experiments by measuring the stained cell nuclei using ML from the LSCM images. According to the preliminary experiments, cell proliferation depended on the size of the mat fibres. Mats B and C had the same fibre diameter, and their cell activity was also quite similar. Mat A showed the most favorable dynamics of proliferative activity and the largest fibre diameter.

Fibroblasts were cultured for seven days to evaluate cell growth on the electrospun mats. After static seeding on the first day, the cells were attached well to both the mat and the culture dish. However, cell growth was higher on all mats except mat D compared with the culture dish (Figure 3b). The primary human skin fibroblasts grew well on mats A, B, and C, while on mats B and C, they demonstrated the highest rates of proliferation (see Figure 3c). During long-term cultivation, fibroblasts were distributed over the surface of the mat and infiltrated them. On the seventh day of cultivation, the surface of the nonwoven material was completely covered with primary human fibroblasts.

The AAV-transduced cells on mat B had similar levels of proliferative activity, as shown in (Figure 3g) and (Figure 3k). The number of transduced cells on mat B increased linearly in all groups (Figure 3d). However, AAV transduction had toxic effects on the cells. On day 7, the cells transduced with AAV-GFP showed a decrease in their proliferation rate, possibly due to the cellular toxicity of AAV or due to the negative effects of GFP, as previously shown [27]. In contrast, the transduction by AVV containing ND4 resulted in a proliferation rate that was comparable to the wild-type cells on the cultural plate. This implies a potential positive effect of ND4 expression on cellular function. According to SEM analysis, on mat B, there were no significant differences in nuclear area variance between the GFP-transduced cells and ND4-transduced cells. On day 7, multiple filopodia running in different directions were observed in all groups.

Cytotoxicity assessment of the electrospun mats (see Figure 3f) showed no significant variation in human skin fibroblast viability when comparing individual mats with the culture plastic. In terms of the number of living cells per nanofibre mat and viability, mat B showed the most satisfactory results. There was no significant difference in the number of live cells between mat B and the control samples. However, the total cell count showed significantly different pairs in the B-GFP and B-ND4 cases according to one-way ANOVA.

The aim of our study was to test whether the area of the nuclei could be used to estimate cell migration through fibrillar surfaces into the nonwoven volume. We also analysed the effects of cell transduction on cell behaviour on the mats. Of significant interest is the study of the topography of substrates for cultivation and its influence on variations in nuclear morphology and the rates of cell proliferation and differentiation [28]. The morphology of the stress fibres of the cytoskeleton plays a crucial role in the deformation and division of the cell nucleus. This phenomenon is directly intertwined with the cellular mechanotransduction signaling and gene regulatory mechanism during cell interaction with the microenvironment [29]. Fibroblast reprogramming can be enhanced using specific cues from the three-dimensional substrate [30]. We found that the area and morphology of the nuclei on the surface and within the nonwoven substrate could fluctuate [31].

Each graph showed a minimum of 15,000 nuclei distributed log-normally. The changes in cell volume and nuclei morphology triggered several intracellular signalling pathways [32]. The nuclei on the culture plate (Figure 3g,k) showed a consistent increase in nuclei area. By day 7, the major-to-minor axis ratio of the nucleus should have been 2–2.5, compared with the 1:1 ratio on day 1. The nuclear area increased, and the diameter ratio increased due to the high proliferative activity of the cells. The different passages may explain the observed difference in nuclear area between experiments g and k on the cultural plate. The cultured primary fibroblasts carrying m.11778G>A on the nonwoven mats exhibited a notable decrease in nuclear area, as demonstrated in the experiment series (Figure 3h,m) compared with the culture plastic (Figure 3h,k). The area of the cell nuclei decreased (Figure 3l,m) on the nonwoven A and B substrates with diameters (Figure 2a) due to the three-dimensional topology of the mats and the hindrance of actin polymerisation. Nevertheless, the total cell area (Figure 3c) increased, which is intriguing. Actin polymerisation promotes morphological transformations in cell proliferation during the attachment procedure [33]. Three factors, namely the highly coiled state of the nuclei in detached cells, pressure differences on the nuclear envelope, and mechanical effects from the cytoplasm, are responsible for the decrease in nuclear volume or area during cell detachment [34]. The fibre topology and morphology influence cell division and drug release. For example, in thin fibres, the release of drugs is accelerated. We used type-B gelatin as a useful model substance to modify the fibre surface topology. The release time of 80% of the gelatin was about 4 days.

The size of the nucleation area remained constant on nonwoven substrates C and D (Figure 3n,o), although their shapes and diameters differed significantly from A and B (Figure 2a). In contrast, the nuclear area of the AAV-GFP-transduced cells fluctuated. The nuclear area decreased by day 4 and increased by day 7 compared with the cells on mat B. The cells transduced with AAV-ND4 were expected to restore ND4 gene function, which would return the cells to a control phenotype with normal functional activity. When comparing the ND4-transduced cells on mat B, we found that their nuclear dynamics were similar to those of the cells on mats C and D (Figure 3n,o). However, the presence of young cells observed on mat B (Figure 3g) indicates that the fibre topology may also contribute to providing a favorable spatial configuration for the cells. Nevertheless, this does not conclusively establish a correlation between the nuclear area, substrate spatial configuration, and cell cycle.

Despite research on various techniques and biological applications of nonwoven polymer composites, only a minority of scientists have investigated lateral cell migration on non-native structures. Therefore, we propose using machine learning tools for counting and segmenting diverse structures, as well as assessing cell migration.

### 3.3. Model of Cell Migration on Three-Dimensional Systems

**Figure 4:**
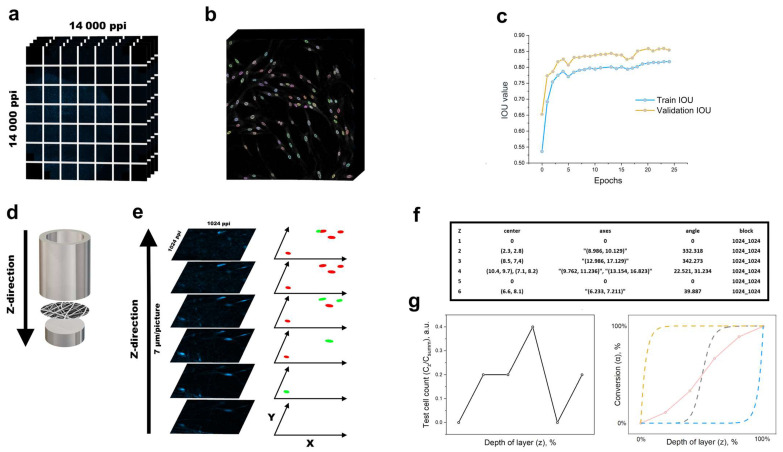
(**a**) Laser-scanning confocal image. (**b**) Part of validation image U-net + aMT. (**c**) IOU score. (**d**) Scanning in z direction on mat holder. (**e**) SSIM per layer. (**f**) Metrics for nuclei. (**g**) Cell count per layer and conversion plot.

The movement of various cell types in response to the microenvironment has been previously addressed [35,36]. The validation IOU value aids in estimating the model’s performance on fresh, novel data. The more the validation IOU value approximates to one, the better the model generalises pre-existing knowledge and detects objects in new images (Figure 4c). We generated a summary image of the dataset layer by layer, with a step of 7 µm for each mat in the z direction (Figure 4d). Each layer was divided into blocks of multiples of 1024 × 1024 pixels to count the nuclei of the primary fibroblasts. Objects detected in focus were entered into a CSV file. Morphological transformations were applied to each image layer to segment the nuclei into relevant metrics (Figure 4f). The SSIM metric was used to compare each nucleus. Only the fibroblast nucleus in focus was assigned to a specific layer (Figure 4e), and it was not counted in subsequent layers. The fibroblast nuclei that were found were encircled with different colours for visualisation (Figure 4b).

As mentioned above, we could procure the coordinates of nuclei and their corresponding measurements for each layer (Figure 4f) by analysing the test images (Figure 4e, from bottom to top). The results indicate a detection of five nuclei in six test images. This ratio of cell numbers in each layer provides reliable insight into the behaviour of the chosen culture on the nonwoven substrate.

The data are displayed on a graph with a *y* axis (C_z_/C_summ_) and *x* axis (z). C_z_ represents the number of cells per layer, and C_summ_ represents the total number of cells in the material (refer to Figure 4g, left). Conversion curves are frequently employed to illustrate the kinetics of n-order model processes in the DSC technique applied at different heating rates [37,38]. The conversion value (α) indicates the fraction of cells in the material layer in this scenario. It corresponds to the red curve in the test data (Figure 4g, right). The cells will settle on the mat holder’s upper layers of the nonwoven substrate. Ideally, the conversion curve should correspond to the yellow dotted exponential curve in (Figure 4g, right). As the mass of cells shifts within the material under examination, the curve should take the form of a blue dashed exponential curve. In general, the cells on the surface remain active and do not disappear from observation if the nonwoven material is spatially positioned in a suitable configuration. In the case of electrospun materials, the free volume fraction typically ranges from 23 to 93%, which correlates with Feigenbaum’s second constant per layer [39,40,41]. As a result, the cell mass conversion curve assumes a Hill function form, as indicated by the grey dashed curve on the right-hand side of Figure 4g.

**Figure 5:**
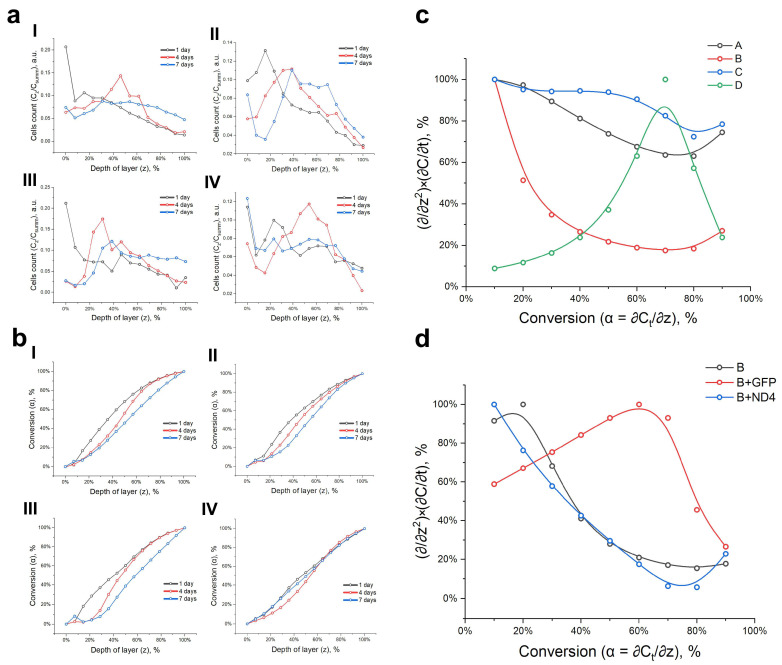
(**a**) Number of cells in each layer per mat. (**b**) Conversion curves of cells per mat. (**c**) Cell migration plot per mat. (**d**) Plot of transduced cell migration on mat B.

Nonwoven materials have characteristics similar to the skin frame in terms of fibre diameter and the presence of free volume for migration and cell colonisation [42]. Figure 5a (i = A, ii = B, iii = C, and iv = D) provides the cell counts for each layer of nonwoven materials of varying compositions. The primary fibroblasts migrated freely in the mats along and between the fibres of compositions A, B, and C. Each data point was generated from three independent prepared samples. On day 7, there was an increase in the number of cells in the upper layers of material D. This observation of the cell dynamics is significant for further justification of the approach.

The number of cells for each composition and diameter distribution increased exponentially, as shown in the graphs for day 1. These conversion curves (Figure 5b, where i = A, ii = B, iii = C, and iv = D) illustrate this information clearly. As the cell mass increased in the deeper layers, the curve took on the shape of a hill. Based on the proliferative activity plot (Figure 3b,d), this indicates the rate of cell division in a specific microenvironment. The rate was assessed from the repopulation time to day 1 (t_1_), from day 1 to day 4 (t_2_), and from day 4 to day 7 (t_3_). To establish the value on the *x* axis (z) for each day’s conversion curve, with 10% α increments, we had to multiply it by the proliferation rate (C_summ_/t_d_) for each location. We plotted the curve on the axes of (α/z^2^) × (C_summ_/t_d_) (α), performed a linear fit, and determined the tangent of the slope (1/z^2^) × (C_summ_/t_d_) for each step. Subsequently, we plotted the obtained curves for the mats of all compositions (Figure 5c).

This unusual format can demonstrate the distinct characteristics of diverse cell cultures on nonwoven substrates, suggest a method for evaluating cell migration potential in various materials, and serve as a valuable tool in assessing migration potential in diverse materials. It can be inferred from the graph that half of the cells in sample composition B accounted for approximately 75% of the migration activity [(1/z^2^) × (C_summ_/t_d_)]. The study of cell migration on distinct nonwoven materials can be likened to the study of cells stimulated by ND4, which can improve functional activity, migration, and homing in the presence of nanofibres (Figure 5d). The graph demonstrates comparable migration activity between cells with gene dysfunction and cells transduced with the ND4 transgene. The graphs demonstrate how cells behave on a nonwoven material for gene transfer efficacy. This includes their proliferation activity per day, which is dependent on the cell density and is based on a significant amount of data.

A study using a CNN found differences between the cellular dynamics of the ND4 and GFP transduced cells. Restoring ND4 expression normalises cell mitochondrial function, which restores the cell distribution, becoming similar to that of wild-type cells. In addition, these studies will allow us to study the effect of AAV-mediated transduction and transgene expression on cell division, cell viability, and the study of the cellular immune response, differentiation, and functional activity [43,44].

## 4. Conclusions

### 4.1. Fibroblast Mechanobiology

Our findings enhance the comprehension of fibroblast mechanobiology. Furthermore, our deep learning-based migration evaluation method has the potential to forecast and regulate cell–substrate interactions, which are extensively utilised in the regenerative medicine and tissue engineering fields. CNNs have surpassed human accuracy in image classification. It is believed that CNNs will greatly impact research on feature and pattern detection in tissue repair tasks.

Numerous rare skin diseases require approbation of therapies on biomimetic systems. In order to achieve optimal therapeutic outcomes, topical gels with different drug delivery methods and substrates coated with autologous or allogeneic cells should take into account the microenvironmental behaviour under different external factors. The article presents a method of evaluating cell migration and cell homing potential, specifically with primary fibroblasts as an example. One approach to assessing cell behaviour involves analysing the nucleus area with a sufficiently large dataset to achieve statistical significance. Studying the behaviour of biological systems in artificial environments could aid researchers in developing multidimensional systems to replace various tissues and organs.

### 4.2. Conclusion and Future Perspectives

In the future, this research will be repeated on fibroblasts with diverse mutations, as well as animal fibroblasts. We will scrutinise the consequences of distinct transgene expressions on cell phenotypic traits simultaneously. Thus, further research can provide valuable insights for improving corrective techniques and implementing advanced CNN methodologies to treat orphan diseases.

## Figures and Tables

**Table 1:** Stress–strain values for each of the incubated mats.

	σ_break_	σ_σ_	ε_break_	σ_ε_
1 h	1.75	0.23	2.81	0.68
2 h	1.39	0.25	2.86	0.22
4 h	1.14	0.24	2.03	0.41
6 h	0.83	0.12	1.67	0.40
24 h	1.04	0.07	2.27	0.20
Control + UV	1.03	0.43	2.54	1.13
Control	1.18	0.40	10.17	1.19

## Data Availability

Data are contained within the article and Supplementary Materials; Training dataset: https://drive.google.com/file/d/1lEWe5-SMsUIyQ6YiYEAbwusTY22_XOQb/view?usp=sharing Available until 25 November 2024.

## Supplementary Materials

The following supporting information can be downloaded at: https://www.mdpi.com/article/10.3390/polym16091187/s1, Figure S1: Supplementary Image 1.
