# Supplementary figures and images for: Deep Learning for Cell Migration in Nonwoven Materials and Evaluating Gene Transfer Effects following AAV6-ND4 Transduction
Source: Polymers (Basel). 2024 Apr 24;16(9):1187. doi: 10.3390/polym16091187 (PMC11085928; doi:10.3390/polym16091187)

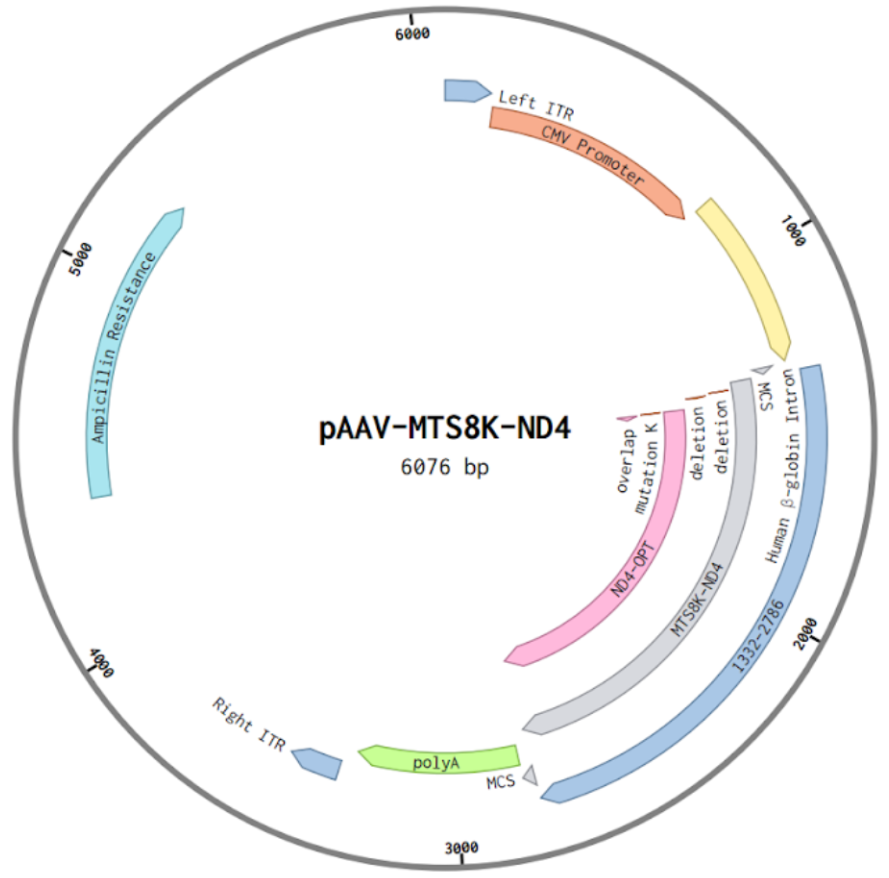

Supplement: Supplementary file 1 [file polymers-16-01187-s001.zip › polymers-2962267-supplementary.png]
